# Prevalence of Adolescent Pregnancy in A Tertiary Care Hospital

**DOI:** 10.31729/jnma.4573

**Published:** 2019-08-31

**Authors:** Shanti Sunuwar Subedi, Sajjan Sharma, Munjal Yadav

**Affiliations:** 1 Department of Obstetrics and Gynaecology, Nobel Medical College and Teaching Hospital, Biratnagar, Nepal

**Keywords:** *contraceptives*, *delivery*, *teenage pregnancy*

## Abstract

**Introduction:**

WHO defines adolescent pregnancy as any pregnancy from a girl who is 10-19 years of age, the age being defined as her age at the time the baby is born. Globally, adolescent birth rate is 44 per 1000 women aged 15-19 years whereas 33 per 1000 in South East Asian region. The main objective of the study is to find the prevalence of adolescent pregnancy in a tertiary care hospital.

**Methods:**

A descriptive cross-sectional study was conducted in Department of Obstetrics and Gynecology, Nobel Medical College and Teaching Hospital, Biratnagar over a period of six months from 1^st^ January 2018 to 30^th^ June 2018. Using the convenient sampling technique, 2688 samples were studied and the descriptive statistical analysis was done.

**Results:**

The prevalence of teenage pregnancy was found to be among 143 (5.3%) [5.3%±0.85% at 95% Confidence Interval]. Maximum adolescents of 95 (66.4%) presented at gestational age between 37 to 40 weeks. Regarding contraception, only 2 (1.4%) of teenage mothers had used Injectable Depo Provera.

**Conclusions:**

Adolescent pregnancy in developed countries is usually outside of marriage and carries a social stigma but in the context of developing countries it usually happens within marriage and half of them are planned reflecting educational status and contraception knowledge. Effective interventions need to be developed like strict enforcement of laws prohibiting teenage marriage.

## INTRODUCTION

WHO defines adolescent pregnancy as “any pregnancy from a girl who is 10-19 years of age”, the age being defined as her age at the time the baby is born. Globally, adolescent birth rate is 44 per 1000 women aged 1519 years whereas 33 per 1000 in South East Asian region. It has been projected that in 2015-2020, more than 1 in 25 adolescent girls aged 15-19 years will give birth.

Adolescent pregnancy has increased rapidly due to unprotected sexual activities, early marriage, illiteracy, low socioeconomic status, poor awareness about contraceptive need and choices.

This study aims to find out the prevalence of adolescent pregnancy in a tertiary care hospital of Nepal.

## METHODS

This descriptive cross-sectional study was conducted in Department of Obstetrics and Gynecology, Nobel Medical College and Teaching Hospital, Biratnagar over a period of six months from 1^st^ January 2018 to 30^th^ June 2018. Ethical clearance was taken from the

Institutional Review Committee. The sample size was calculated as per below formula:

Sample size (n)=Z^2^ × pq/e^2^

     =(1.96)^2^ × 0.5 × (1-0.5) / (0.02)^2^

     =2401

where,

Z= 1.96 for confidence interval at 95%
p= prevalence, 50%
q=1-p
e= margin of error, 2%

Keeping non-response rate at 10%, the final sample size is 2641. Convenient sampling technique was applied. Total 2688 samples were taken for the study to minimize the possible biases (information bias and interviewer's bias) and to make it more scientific.

All pregnant women who delivered baby in this hospital during study duration were included in the study after their consent. Cases planned for medical termination of pregnancy were not included. Information was noted in a well-structured proforma.

Maternal outcomes recorded were anemia, preterm delivery, hypertensive disorders in pregnancy, operative deliveries with indications of caesarean section, postpartum complications and maternal mortality. Perinatal outcomes recorded were low birth weight, stillbirth, indications of neonatal admission and neonatal death. The data were entered in Microsoft Excel and analyzed using SPSS software version 23.

## RESULTS

Out of the 2688 deliveries, the prevalence of adolescent pregnancy was 143 (5.3%) [5.3% ±0.85% at 95% CI]. Mean age was 18.4 years (ranges from 16 to 19 years). Out of them, 57 (39.9%) had early marriage at the age of 18. Most of the adolescents were unbooked, from low socioeconomic status and 129 (90.2%) were primigravida. Maximum adolescents of 95 (66.4%) presented at gestational age between 37 to 40 weeks and postdated pregnancy was 28 (19.6%). Among them, the caesarean section was done in 42 (29.4%) (Table 1).

**Table 1:** Mode of Delivery

Mode of delivery	n (%)
Cesarean section	42 (29.4)
NVD	94 (65.7)
Instrumental (Vacuum)	7 (4.9)
Total	143 (100)

**Figure 1.**
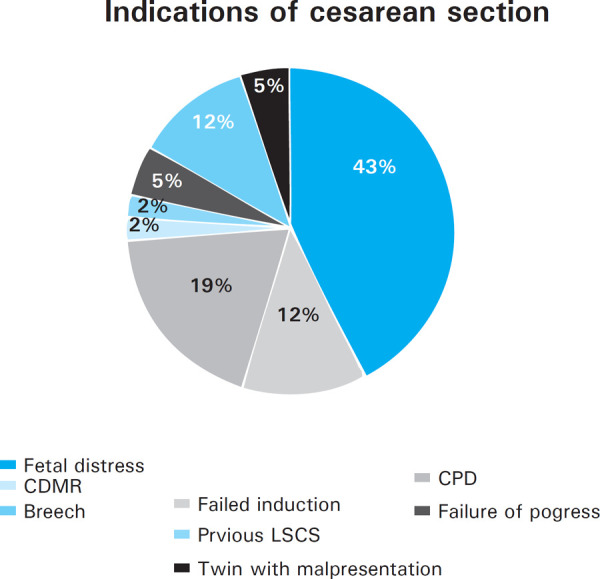
Indications of caesarean section.

The most common indication for cesarean section was fetal distress in 18 (43%), Cephalopelvic Disproportion (CPD) in 8 (19%) followed by failed induction in 5 (12%) (Figure 1).

About 67 (46.8%) of adolescents mothers had maternal complications. The most common complication was Premature Rupture of Membrane (PROM) in 20 (14%) followed by anemia in 19 (13.3%), hypertensive disorders in pregnancy were observed in 9 (6.29%) and there was no maternal mortality in the study period (Table 2).

**Table 2:** Antepartum complications.

Antepartum complications	n (%)
Anemia	19 (13.3)
APH	1 (0.6)
PIH	4 (2.7)
Preecclampsia	3 (2.0)
Ecclampsia	2 (1.39)
GDM	4 (2.7)
CPD	7 (4.9)
Preterm labor	4 (2.7)
PPROM	3 (2.0)
PROM	20 (14.0)
None	76 (53.1)

Regarding contraception, only 2 (1.4%) of teenage mothers had used Injectable Depo Provera. The mean birth weight of newborn was between 2.5 to 4 kg, 117 (81.8%) being majority appropriate for the gestational age i.e. 2.5 kg or more. Only 25 (17.5%) were low birth weight whereas 20 (14%) accounting for preterm deliveries. There was one case of stillbirth and 22 (15.4%) were admitted in NICU for Meconium Aspiration Syndrome (MAS), birth asphyxia, respiratory distress syndrome and Transient Tachypnea of Newborn (TTN) (Table 3 and 4). Out of babies born alive, 10 (7%) had APGAR score of less than 7 in 5 minutes.

**Table 3:** Postpartum complications.

Postpartum complications	n (%)
Prolonged labour	1 (0.69)
PPH	1 (0.69)
None	141 (98.60)

**Table 4:** Perinatal complications.

Perinatal complications	n (%)
Low birth weight	25 (17.5)
Preterm	20 (14)
NICU admission	22 (15.4)
Still birth	1 (0.7)

## DISCUSSION

Adolescent pregnancy is a public health problem and this period is a distinct and unique physical and developmental stage in woman's life. Currently 40% of the population in Nepal is under the age of 18 years (Demographic Changes of Nepal: Trends and policy implications, 2017). In Nepal, 17% of adolescent aged 15-19 are already mothers or pregnant with their first child. The prevalence in Nepal have been reported varyingly. The prevalence of teenage pregnancy in the current study is 5.3% which is similar to 5.10% in Yasmin et al.^8^ Similarly, results that of Subedi A. et al^5^ in 2018 showed incidence of 6% and Suwal A. et al in 2012 showed 6.85% but 2.81% higher than Seneesh KV et al. The incidence of teenage pregnancy is highest in Bangladesh 35% followed by Nepal 21% and India 21% in the context of South Asia. However, in contrast to this study the incidence of teenage pregnancy was as high as 9.7% by Kayastha et al.

According to NDHS 2016, the median age at first marriage for women age (25 to 49 years) is 17.9 years. Women initiate sexual activity at the same time of marriage at age 17.9 years. More than half (52%) of women are married by age 18. In our study, meanage of marriage was 18.4 years (ranges from 16 to 19) and majority of adolescent mothers (52.4%) were at 19 years of age similar to that of Subedi et. al.^5^

Most of the teenagers in the study were unbooked, from low socioeconomic status reflecting poverty and poor prenatal care. Majority of them were primigravida 90.2% as expected. Regarding the mode of delivery 65.7% underwent normal vaginal delivery and 29.4% of them delivered by cesarean section. Findings similar to this study have also been shown by studies of Mukhopadhyay P. et al. in 2010.^11^ The higher proportion of normal vaginal delivery could be due to smaller babies (17.4%) in this study.

Several studies from Nepal have documented poorer outcomes for children born to adolescent mothers compared to older mothers. The most common indication of cesarean delivery in this studywasfetal distress followed by CPD similar to the findings of Yasmin et al.^8^ and Mukhopadhyay P. et al.^11^ One of the proposed explanations for these adverse birth outcomes are biological immaturity. Teenage pregnancy has emerged as a burden to the social infrastructure due to its catastrophic consequences. Teenage mothers were nearly three times more at risk of developing anemia and delivering preterm. Derme and his colleagues reported that PROM was the most common maternal complication which is similar to our study.^13^ In this study,13.3% patients were anemic and 14% had preterm delivery. Only 1.4% of teenage mothers used the temporary methods of contraception which could be the reason for early childbearing in teenagers. There was no case of maternal mortality in our study. 17.5% of teenage mothers had low birth weight which is similar to the findings reported by Yasmin et al. (16.86%).^8^ Swati Mahajan reported the incidence of LBW to be 19%.^14^ There was one case of stillbirth and babies of 15.4% of teenage pregnancy were admitted in NICU to rule out MAS followed by birth asphyxia, respiratory distress syndrome and TTN.

The primary limitation of this study was that since this study was conducted in a peripheral tertiary-care hospital and there were maximum chances of referred cases and it might not truly reflect the current situation in community basis. Another important aspect is that the exact quantification of adverse perinatal outcomes of teenage pregnancy might have been biased due to unequal distribution of sociodemographic characteristics and also because the delayed complications that can occur beyond 48 hours could not be observed because of inadequate follow up protocol.

## CONCLUSIONS

The present study attempts to emphasize on adolescentfriendly health care services including regular antenatal checkup, rest, adequate sleep and appropriate counseling. Teenage pregnancy characterizes a high risk group because of the double burden of reproduction and growth and is associated with social issues including lower educational levels and poverty. Adolescent pregnancy in developed countries is usually outside of marriage and carries a social stigma but in the context of developing countries it usually happens within marriage and half of them are planned reflecting educational status and contraception knowledge.

Effective interventions need to be developed like strict enforcement of laws prohibiting teenage marriage. Contraceptive practice need to be promoted in order to delay future pregnancy and we need to do lot in this field to improve the contraceptive prevalence rate in Nepal.

## Conflict of Interest:

**None.**

## References

[1] World Heath Organization (2004). Adolescent pregnancy-issues in adolescent health and pregnancy.

[2] United Nations (2017). World population prospects: the 2017 revision.

[3] Ministry of Health (2016). Nepal Demographic and Health Survey.

[4] Yasmin G, Kumar A, Parihar B (2014). Teenage Pregnancy - Its Impact On Maternal And Fetal Outcome. Int J Scientific Study.

[5] Subedi A, Shrestha J, Shrestha A, Gurung S (2018). Maternal and perinatal outcome of teenage pregnancy in a tertiary care centre. Nep J Obs Gynae.

[6] Suwal A (2012). Obstetric and perinatal outcome of teenage pregnancy. J Nepal Health Res Counc.

[7] Seneesh KV, Shah M (2015). Feto - Maternal Outcome in Teenage Pregnancy - A Comparative Case Control Study. J Preg Child Health.

[8] The Word Bank Children and Youth Committee (2005). Children and Youth: A Resource Guide for World Bank Staff.

[9] Kayastha S, Pradhan A (2012). Obstetric outcome of teenage pregnancy. Nep J Obs Gynae.

[10] Sharma V, Katz J, Mullany L, Khatry S, LeClerg S, Shrestha S (2008). Young maternal age and the risk of neonatal mortality in rural Nepal. Arch Pediatr Adolesc Med.

[11] Mukhopadhyay P, Chaudhuri RN, Bhaskar P (2010). Hospital-based perinatal outcomes and complications in teenage pregnancy in India. J Health Popul Nutr.

[12] Mahavarkar SH, Madhu CK, Mule VD (2008). A comparative study of teenage pregnancy. J Obs Gynae.

[13] Derme M, Leoncini E, Vetrano G, Carlomagno L, Aleandri V (2013). Obstetric and perinatal outcomes of teenage pregnant women: a retrospective study. Epidemiology Biostatistics and Public Health EBPH.

[14] Mahajan S (2007). Teenage deliveries and risk of adverse outcome. A hospital based case-control study.

